# Coronary Microvascular Dysfunction in Cardiomyopathies: Insights on Clinical and Prognostic Roles

**DOI:** 10.31083/RCM46829

**Published:** 2026-02-26

**Authors:** Federico Giacobbe, Pierre Meynet, Marco Balducci, Sergio Capoccia, Rino Andrea Cimino, Arianna Morena, Antonio Dalu, Fabrizio D’Ascenzo, Ovidio De Filippo, Filippo Novarese, Francesco Bruno, Claudia Raineri, Federico Conrotto, Athanasios Sakalidis, Pierluigi Omedé, Giuseppe Giannino, Filippo Angelini, Pier Paolo Bocchino, Veronica Dusi, Italo Porto, Gaetano Maria De Ferrari

**Affiliations:** ^1^Division of Cardiology, Cardiovascular and Thoracic Department, Città della Salute e della Scienza, 10126 Turin, Italy; ^2^Division of Cardiology, Department of Medical Sciences, University of Turin, 10126 Turin, Italy; ^3^Department of Cardiology, Research, Education and Development Division, Royal Brompton and Harefield Hospitals, SW3 6NP London, UK; ^4^Division of Cardiology, San Martino Hospital, University of Genoa, 16132 Genoa, Italy; ^5^Department of Cardiology, Hippokration Hospital of Athens, 11527 Athens, Greece

**Keywords:** coronary circulation, microcirculation, microvascular angina, cardiomyopathies

## Abstract

Coronary microvascular dysfunction (CMD) is a key driver of ischemia and prognosis across several non-ischemic cardiomyopathies. This review summarizes the main tools for diagnosing microvascular dysfunction and available evidence on CMD incidence and the prognostic role in patients with cardiomyopathies. In dilated cardiomyopathy, CMD is associated with reduced myocardial blood flow, greater fibrosis, adverse remodeling, and worse outcomes. In hypertrophic cardiomyopathy, CMD is highly prevalent and multifactorial (arteriolar remodeling, reduced capillary density, extravascular compression, diastolic dysfunction, and/or left ventricular (LV) outflow obstruction), correlating with fibrosis, heart failure, and arrhythmias/sudden death. In Takotsubo syndrome, CMD appears acute and reversible, with microvascular spasms as a predominant mechanism and plausible pathophysiologic basis of the event. In arrhythmogenic right ventricular cardiomyopathy, preliminary data show a blunted hyperemic response and autonomic abnormalities that may impair microvascular vasodilation. In infiltrative and storage diseases (amyloidosis and Anderson–Fabry disease), CMD is often early, preceding hypertrophy/fibrosis, and contributes to symptoms, contractile dysfunction, and adverse outcomes; in sarcoidosis, microvascular inflammation reduces coronary flow reserve (CFR) and is associated with events. Targeted therapies remain limited; optimization of risk factors and drugs that modulate endothelial/metabolic function (statins, angiotensin converting enzyme (ACE) inhibitors, vasodilating β-blockers, calcium channel blockers, sodium glucose cotransporter 2 (SGLT2) inhibitors) yielded variable signals; device-based and nonpharmacologic strategies are under investigation. In conclusion, integrating microcirculatory assessment improves risk stratification and may furnish future therapeutic targets across cardiomyopathies.

## 1. Introduction 

Coronary microvascular dysfunction (CMD) and epicardial/microvascular vasospasm are the primary pathophysiological mechanisms in patients affected by ischemia with non-obstructive coronary artery disease (INOCA) [1]. This is a non-negligible condition, which is estimated to affect approximately 3–4 million individuals [2], with a significantly higher prevalence among women [3].

Currently, CMD poses a diagnostic challenge for clinicians, causing a remarkable reduction in quality of life for patients and increased healthcare costs [4]. In recent times, studies have broadened their focus beyond refractory angina to include other conditions, since microvascular obstruction (MVO) has shown a relevant role in determining the severity of symptoms and prognosis in patients affected by cardiomyopathies.

The 2023 European Society of Cardiology guidelines define cardiomyopathies as myocardial disorders in which the heart muscle is structurally and functionally abnormal, in the absence of coronary artery disease (CAD), hypertension, valvular disease and congenital heart disease. As being a heterogenous group of entities, several phenotypes have been distinguished according to the morphological and functional features of the disease. CMD is being studied in this setting given its relevant role, regardless of the mechanisms leading to microvascular dysfunction.

### 1.1 Classification and Pathophysiology

CMD affects the pre-arterioles, arterioles and capillary networks and is classified into two main forms, structural and functional. Structural CMD is characterized by pathological remodelling of the arteriolar architecture, primarily resulting in a reduction of vasodilatory capacity due to fixed anatomic alterations, independently from endothelial function. This may occur as a primary condition or in association with structural heart diseases such as hypertrophic or dilated cardiomyopathy, and it is frequently associated with systemic comorbidities, including chronic kidney disease and diabetes mellitus [5]. Key histological features of structural CMD include increased arteriolar wall thickness, enhanced wall-to-lumen ratio, vascular smooth muscle cell proliferation, perivascular fibrosis, intimal thickening, and microvascular rarefaction.

**Fig. 1.**
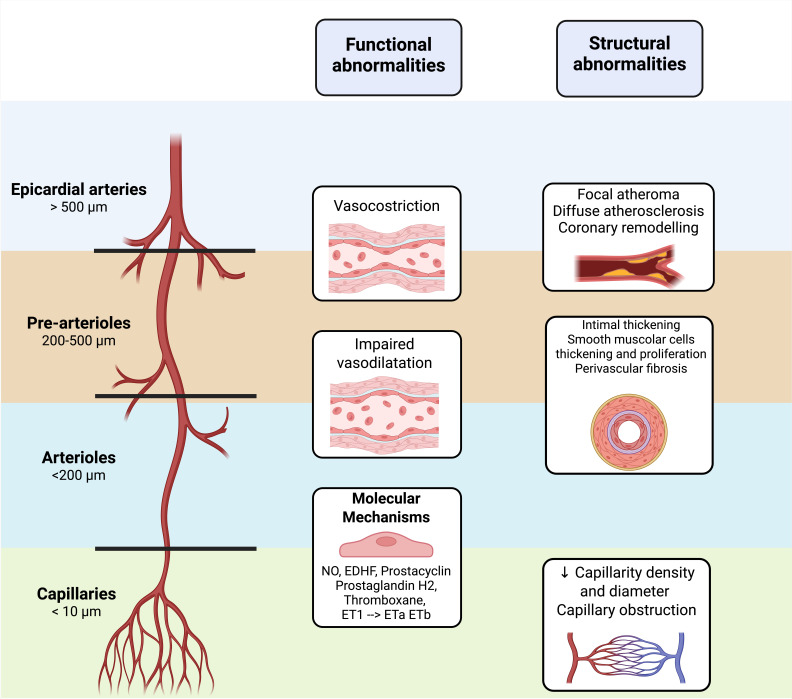
**Coronary microvascular dysfunction (CMD) results from either functional or structural abnormalities of the coronary microcirculation**. Functional CMD is characterized by impaired vascular homeostasis, due to reduced availability of vasodilators (nitric oxide, endothelium-derived hyperpolarizing factors (EDHF), prostacyclin) and/or increased vasoconstrictor activity (thromboxane and endothelin-1 receptor activation). Structural CMD reflects pathological remodeling of the microvasculature, with reduced capillary density and lumen diameter caused by intimal thickening, smooth muscle cell proliferation, and perivascular fibrosis, typically related to atherosclerosis or primary myocardial disease. The downward arrow is referred to the reduction of capillary density and diameter. Created with BioRender.

Functional CMD is primarily characterized by endothelial dysfunction, arising from an imbalance between vasodilatory and vasoconstrictive factors, leading to a disruption of vascular homeostasis. This condition is primarily driven by an overproduction of vasoconstrictor factors such as endothelin-1, superoxide, hydrogen peroxide, and thromboxane that override normal vasodilatory mechanisms, contributing to microvascular spasm and impaired vascular tone regulation [6]. Under physiological conditions, increased myocardial oxygen demand, such as during exercise, triggers coordinated endothelium-dependent vasodilation in both the epicardial and microvascular coronary circulation, primarily mediated by nitric oxide (NO) and prostacyclins [1, 7]. In the setting of endothelial dysfunction this response is blunted or paradoxically replaced by vasoconstriction, further impairing myocardial perfusion [7]. The main pathophysiological abnormalities are summarized in Fig. 1.

As a matter of fact, the diagnosis of CMD is given through measurement of coronary flow reserve (CFR), which is defined as the ratio between the myocardial blood flow (MBF) at maximal hyperaemia and the basal one. A CFR less than 2.5 is indicative of microvascular disease, while an assessment of the index of microvascular resistance (IMR) is essential to distinguish between structural and functional types [8]. Based on a combined analysis of CFR and IMR, CMD is classified as structural (CFR <2 and IMR >25), functional (CFR <2 and IMR <25) or compensated structural (CFR >2 and IMR >25).

### 1.2 Non-Invasive Assessment of CMD

Microvascular dysfunction may be assessed through non-invasive tools, which help to confirm the disease quickly and safely. In this sense many non-invasive tools may be exploited: transthoracic doppler echocardiography (TTDE), single photon emission computed tomography (SPECT), positron emission tomography (PET) and stress perfusion cardiac magnetic resonance (CMR).

Despite the low sensitivity (44%) and specificity (56.1%) [9], TTDE may detect CMD through stress-induced abnormalities in regional wall motion, as measured by the wall motion score index (WMSI), as well as in regional systolic wall thickening.

Ultrasound contrast agents considerably enhance the sensitivity of this tool and their passage through the myocardium enables the assessment of myocardial perfusion, thus improving the detection of microvascular disease. Coronary flow reserve velocity can be calculated from the flow velocity recordings at rest and during stress in the left anterior descending artery, ensuring a high safety and optimal concordance with invasive CFR measurements.

SPECT and PET provide information about regional MBF according to the amount of retention of radionuclides. PET allows robust absolute quantitative measures of MBF with superior image quality and lower radiation exposure as compared to SPECT. Furthermore, PET permits the calculation of myocardial flow reserve defined as the ratio of stress to resting MBF and considered to be abnormal for values below 2.0.

Stress perfusion CMR may assess microvascular dysfunction by means of either visual, semi-quantitative or quantitative adjudication. Visual assessment first relies on the evaluation of circumferential subendocardial perfusion defect. However, the semiquantitative and quantitative perfusion assessments have proven more reliable for diagnosing CMD [10]. The semi-quantitative method is based on the assessment of myocardial perfusion reserve (MPR) index, which offers a good correlation with coronary reactivity testing for CMD [11]. Conversely, quantitative myocardial perfusion assessment is based on evaluation of MPR, defined as the ratio between MBF during hyperaemia and MBF at rest, reduction of stress MBF and alteration of endocardial to epicardial perfusion ratio (endo/epi ratio), the latter representing a transmural maldistribution of MBF during stress.

The main limitation of non-invasive techniques is their lower precision, as they reflect an integrated measure of blood flow throughout the entire coronary circulation, including both epicardial and microvascular components. Therefore, conversely to invasive methods, non-invasive techniques are unable to distinguish between the relative contribution of macrovascular and microvascular compartments [8].

### 1.3 Invasive Assessment of CMD

A definitive diagnosis of CMD may only be achieved through invasive evaluation. This is typically performed following coronary angiography, through a physiology assessment performed with a pressure wire, which has been historically validated in the left anterior descending coronary artery, though being now widely feasible and reliable in all major epicardial territories [8].

CFR can be calculated using the following tools:

- Bolus thermodilution: baseline transit time divided by hyperaemic transit time;

- Continuous thermodilution: the ratio between hyperaemic and resting absolute coronary flow;

- Doppler flow velocity: the hyperaemic flow divided by baseline flow velocity.

To measure CFR the interventional cardiologist may use an intracoronary Doppler-tipped guidewire or a pressure/temperature-sensor tipped guidewire. The former uses a diagnostic intracoronary guidewire equipped with a single Doppler sensor on the distal tip, so that the Doppler CFR is obtained through the ratio between the average peak flow velocity (APV) during hyperaemia and the APV at rest [12]. On the other hand, thermodilution-based CFR is measured using a guidewire with pressure and temperature sensors, which can estimate flow velocity from the inverse of the mean transit time (Tmn) of a room-temperature saline bolus.

Microvascular resistances can be assessed by combining pressure and flow data: through thermodilution, the IMR is calculated as the product of distal coronary pressure and hyperaemic Tmn. An IMR >25 is considered diagnostic for increased microvascular resistance [13].

In Doppler-based methods, hyperaemic microvascular resistance (HMR) may also be calculated as the ratio between distal coronary pressure and APV during maximal hyperaemia. IMR and HMR offer a flow-independent quantification of microvascular resistance.

Detection of coronary artery spasm, either epicardial or microvascular, requires intracoronary provocation test, commonly performed using acetylcholine (ACh). In healthy endothelium ACh causes vasodilation via NO release mediated by the muscarinic receptor M1 and M3 in the endothelial cells, which activates the NO synthetase; on the contrary, in dysfunctional endothelium ACh directly stimulates the M3 muscarinic receptors in the smooth muscle resulting in the activation of phospholipase C and subsequent vasoconstriction [14].

In microvascular spasm, patients typically exhibit typical chest pain with ischemic electrocardiographic alterations in the absence of angiographic evidence of epicardial coronary artery spasm. Coronary angiography alone may show remarkable slow flow due to the spastic microcirculation, while modern wire-based systems may prove transient but notable rise in IMR and reduction of Tmn [15].

All the tools for invasive and non-invasive assessment of CMD are described in Table 1.

**Table 1.** **Invasive and non-invasive tools for CMD assessment**.

Invasive assessments					
Modality	Metric	Cut-off		Clinical availability	Utility
Intracoronary Doppler	CFR	<2.5		Currently unavailable	∙ High and extensively validated
	hMR	>2.5			
	Pzf	≥42 mmHg			∙ Recommended in guidelines
	AChFR	≤1.5			
Bolus thermodilution	CFR	<2.5		Widely available	∙ Good, extensive use reported, moderate reproducibility
	IMR	≥25			
	RRR	<3.5			∙ Recommended in guidelines
	MRR	≤3.0			
Continuous thermodilution	CFR	<2.5		Research or academic centres	∙ Increasingly investigated, high reproducibility
	Absolute hyperaemic resistance	>480 Woods Units			
	MRR	<2.1			∙ Recommended in guidelines
Non-invasive assessments					
Modality	Stressor	Metric	Threshold	Clinical availability	Utility in RA
CPET	Exercise	MVO_2_	Peak MVO_2_ 17.3 vs. 27.3 mL/kg/min in normal controls	Research or academic centre	Available but limited evidence
Stress Echocardiography					
Wall motion assessment	Exercise	WMSI	-	Routine	Widely available but limited evidence
	Dobutamine				
	Adenosine				
	Dipyridamole				
LAD Doppler	Adenosine	CFVR	CFVR <2	Research or academic centre	Available and increasing evidence
	Dipyridamole				
MCE perfusion	Adenosine	Refill time	>2 secs during vasodilator stress	Research or academic centre	Investigational
	Dipyridamole	Stress MBF	236 intensity units/sec		
	Regadenoson	Microvascular flux rate (β)	1.6/sec		
Nuclear					
SPECT (^99m^Tc-sestamibi, ^99m^Tc-tetrofosmin)	Adenosine	Qualitative & summed scores	-	Routine	Widely available but limited evidence
	Regadenoson				
PET (^15^O, ^82^Rb, ^13^NH)	Adenosine	CFR	CFR <2	Research or academic centre	Limited availability but recommended
	Dipyridamole				
	Regadenoson				
Stress Perfusion CMR					
Visually adjudicated	Adenosine	Visual perfusion defect	Circumferential subendocardial perfusion defect	Routine	Available but limited evidence
	Regadenoson				
Semi-quantitative	Adenosine	MPRI	1.84	Research or academic centre	Available but limited evidence
	Regadenoson				
Quantitative	Adenosine	MPR	MPR <2.2	Research or academic centre	Limited availability but recommended
	Regadenoson	Stress MBF	Stress MBF <1.82		
		Endo/epi ratio			

AChFR, acetylcholine flow reserve; CFR, coronary flow reserve; CFVR, coronary flow velocity reserve; CMR, cardiac MRI; endo, epi ratio, endocardial to epicardial ratio; hMR, hyperaemic microvascular resistance; IMR, index of microcirculatory resistance; LAD, left anterior descending; MBF, myocardial blood flow; MCE, myocardial contrast echocardiography; mm, millimetres; MPR, myocardial perfusion reserve; MPRI, myocardial perfusion reserve index; MRR, microvascular resistance reserve; PET, positron emission tomography; Pzf, pressure at zero flow; RA, refractory angina; RRR, resistive reserve ratio; WMSI, wall motion score index; SPECT, single photon emission computed tomography; MVO_2_, Myocardial Oxygen Consumption.

## 2. Dilated Cardiomyopathy

Although dilated cardiomyopathy (DCM) is conventionally defined as a non-ischemic myocardial disorder marked by left ventricular enlargement and systolic dysfunction without substantial epicardial coronary artery involvement, growing evidence highlights a pivotal role of CMD, predominantly driven by microvascular obstruction, to its pathogenesis and advancement [16].

Typically observed in the context of acute myocardial infarction reperfusion, MVO is associated with failure of myocardial perfusion even when the epicardial vessels are patent. Its aetiology frequently involves factors such as microembolization, endothelial dysfunction, microvascular spasm, and extravascular compression [17].

In patients with DCM, even in the absence of overt coronary artery disease, hallmark features include reduced CFR and MBF. These abnormalities have been consistently demonstrated using advanced imaging modalities in DCM populations [18].

### 2.1 CMD Pathophysiology in DCM

Multiple mechanisms contribute to microvascular dysfunction in DCM. Chronic inflammation and oxidative stress, common features in the pathogenesis of DCM, promote endothelial dysfunction within the coronary microcirculation, leading to impaired vasodilatory capacity and a shift toward vasoconstriction. The morphological changes observed may be characterized by adverse remodelling of the arterioles, resulting in medial wall thickening (due to smooth muscle hypertrophy and increased collagen deposition) and variable degrees of intimal thickening, causing altered coronary physiology and coronary blood flow [1].

Furthermore, myocardial fibrosis, frequently observed in case of adverse remodelling in DCM, may further compromise microvascular perfusion through extravascular compression and increased myocardial stiffness [19].

To date, several studies have demonstrated an inverse correlation between the extent of myocardial fibrosis detected by late gadolinium enhancement (LGE) on cardiac magnetic resonance and MPR in these patients, suggesting a direct link between structural remodelling and microvascular impairment, driven by abnormal vasodilation and inadequate MBF augmentation during stress [18].

### 2.2 Prognostic Relevance of CMD in DCM

The presence of MVO in DCM is not merely an epiphenomenon but represents a significant contributor to disease progression. MVO may result in recurrent, often transient, subendocardial ischemia, thereby exacerbating myocardial injury, promoting fibrotic remodeling, facilitating ventricular arrhythmogenesis, and worsening heart failure (HF) symptoms [20, 21]. Some evidence suggests that ventricular unloading in DCM patients can lead to an improvement in MVO and IMR [22].

Additionally, in DCM patients the severity of microvascular dysfunction has been linked to the degree of left ventricular (LV) impairment and is an independent predictor of adverse cardiovascular events, including hospitalization for HF and all-cause mortality [16, 23].

Given these findings, understanding and addressing MVO in this context may provide further valuable insights on DCM patients’ prognosis and may represent a critical avenue for future therapeutic interventions.

## 3. Hypertrophic Cardiomiopathy

Hypertrophic cardiomyopathy (HCM) is defined by LV hypertrophy that cannot be explained solely by abnormal loading conditions [24]. Myocardial ischemia pathophysiology in the context of HCM is complex and highly heterogeneous. Although many contributors have been identified, such as myocardial hypertrophy, increased filling pressures, epicardial CAD, anomalous coronary artery anatomy and myocardial bridges, CMD remains the leading cause of myocardial ischemia in HCM. In this setting, CMD arises from a multifaceted interplay of both structural and functional abnormalities of the coronary microcirculation.

### 3.1 CMD Pathophysiology in HCM

A reduced coronary vasodilator reserve in the absence of epicardial coronary artery stenosis is common in HCM, both in hypertrophied and non-hypertrophied LV segments [25]. Coronary blood flow studies in HCM patients have shown a higher rest blood flow, blunted CFR, and lower coronary vascular resistance compared with controls. These findings are thought to reflect a near maximal coronary vasodilatation at rest, required to supply the increased metabolic demands of hypertrophied myocardium, leaving little residual vasodilatory reserve during stress [26].

From an ultrastructural point of view, consistent abnormalities of the small intramural coronary arteries have been described in HCM, including medial hypertrophy, intimal hyperplasia and luminal narrowing [27]. Furthermore, reduced capillary density has been reported in hypertrophied LV segments, with an inverse relationship between the degree of hypertrophy and capillary rarefaction. Beyond these structural alterations, coronary autoregulation is also impaired in HCM, with dampened coronary vasodilator reserve and stress-induced hypoperfusion [25]. Functional factors also encompass some hallmark hemodynamic features of HCM, such as extravascular compression, diastolic dysfunction, and LV outflow tract obstruction (LVOTO). In addition to extrinsic compression of the small vessels mediated by hypertrophied myocytes and fibrosis [28], systolic compression of intramyocardial blood vessels may further disrupt coronary hemodynamic [29]. Given that coronary perfusion predominantly occurs during diastole, diastolic dysfunction may further impair myocardial perfusion through inadequate microvascular decompression [30]. Finally, LVOTO has been associated with reduced perfusion reserve and MBF [31], particularly at the subendocardial level [32], likely due to the increased myocardial workload required to overcome the outflow obstruction.

Interestingly, myocardial perfusion defects, lower MPR and reduced blood flow have been described even in phenotype-negative genotype-positive subjects carrying likely pathogenic or pathogenic sarcomeric variants [33]. Furthermore, phenotype-positive HCM subjects with identified sarcomeric variants display more severe microvascular dysfunction compared with genotype-negative counterparts [34]. Myocardial oxygen demand is increased even in the absence of hypertrophy in HCM carriers, a finding related to an inefficient sarcomere contraction [35]. Some pieces of evidence point to a “pre-hypertrophy myocyte phenotype switching” hypothesis, with vascular smooth muscle hypertrophy mediated by altered genetic expression [28], or to a “myocyte/capillary embryological coupling” hypothesis, whereby microvascular abnormalities precede the development of clinically overt hypertrophy [33].

### 3.2 Prognostic Relevance of CMD in HCM

The natural history of HCM is mainly dominated by the progression to HF and the risk of sudden cardiac death (SCD) [36, 37]. Besides classical and established risk factors, myocardial ischemia has emerged as an important element in risk stratification [38, 39]. Chronic and reiterative ischemic injuries have been associated with replacement fibrosis in HCM, as demonstrated by CMR studies, leading to negative LV remodelling, arrhythmias and SCD [40]. Importantly, while chest pain is common in HCM, myocardial ischemia may also occur in asymptomatic individuals. In this regard, investigation of myocardial ischemia should be considered as an integral part of HCM patient diagnostic work-up, independently from symptoms status.

Reduced stress MBF has been correlated with adverse LV remodelling, chamber dilation, wall thinning, and progression towards systolic dysfunction [41]. Quantitative perfusion assessment using PET, SPECT [42] or stress perfusion CMR [43] has been shown to predict functional class deterioration and HF development. Indeed, a MBF ≤1.1 mL/g/min during stress has proved to predict adverse outcome and cardiovascular (CV) mortality [41]. On the contrary, in a large cohort of HCM patients, qualitative assessment of perfusion defects on CMR was unable to predict HF [44]. Given the typically diffuse nature of perfusion abnormalities in HCM, absolute quantitative perfusion measures appear more suitable for detecting and characterizing CMD.

### 3.3 Role of CMD in Arrhythmogenesis and Sudden Cardiac Death in HCM

Arrhythmogenesis in HCM is multifactorial, stemming from the combination of an abnormal macro- and microscopic substrate, hemodynamic perturbations, rhythm disturbances, intracellular calcium dysregulation and myocardial ischemia [36]. In an autopsy study of 19 subjects with HCM and SCD (age ≤35 years), histological evidence of acute or subacute myocardial ischemia was identified in most of the cases; none had significant epicardial CAD [25]. Myocardial fibrosis has been clearly recognized as a risk factor for SCD [45]. CMR and PET studies have found a correlation between LGE and grade of hyperaemic MBF, suggesting a possible interrelation between LGE extent and ischemia [46]. Of note, even after adjustment for fibrosis, myocardial ischemia has been independently associated with ventricular arrhythmias, although the strength of this association varies across studies [46, 47, 48].

## 4. Takotsubo Syndrome (TTS)

Takotsubo Syndrome (TTS) is an acute and usually reversible acute HF syndrome, characterized by a transient catecholamine-mediated myocardial stunning in the absence of a culprit coronary lesion explaining the LV dysfunction. Given its temporary nature, the 2023 European Society of Cardiology guidelines on cardiomyopathies do not classify TTS as a distinct cardiomyopathy.

The Heart Failure Association diagnostic criteria summarize the features of TTS: (I) transient wall motion abnormalities often — but not invariably — preceded by a physical or emotional stressor; (II) extension of regional wall motion abnormalities beyond a single epicardial coronary distribution; (III) absence of a culprit coronary lesion, hereby including thrombus, dissection or plaque rupture; (IV) new and reversible electrocardiographic abnormalities (ST-T changes, left bundle branch block, T waves inversion, QT prolongation); (V) significantly elevated natriuretic peptide during the acute phase; (VI) positive but relatively modest elevation in serum troponin; (VII) recovery of LV systolic function on cardiac imaging (usually within 3 to 6 months) [49].

TTS occurs more frequently in women and elderly people, the latter being a subgroup of patients at higher risk of TTS-related major complications [50].

### CMD Pathophysiology in TTS 

The causes of TTS are still debated with several etiopathogenetic mechanisms proposed, such as multivessel epicardial coronary spasm, catecholamine-induced myocardial stunning, spontaneous coronary thrombus lysis, and acute microvascular spasm [49]. Regardless of the underlying etiopathogenetic mechanism, the common pathophysiological pattern of TTS seems to involve acute and transient coronary microvascular dysfunction.

The involvement of the coronary microcirculation was first suggested by PET and SPECT imaging studies demonstrating impaired perfusion in regions corresponding to wall motion abnormalities despite the absence of obstructive CAD [51, 52].

Rigo *et al*. [53] reported a reduction of CFR evaluated by dipyridamole echocardiography test in the acute phase (within 24 hours from admission) in a cohort of 30 TTS patients. Reversibility of the microvascular dysfunction was demonstrated by the improvement of CFR assessed at discharge and at 6 months, which interestingly paralleled with an improvement of WMSI.

Galiuto *et al*. [54] compared TTS patients with a control group of 15 patients with ST-elevation myocardial infarction (STEMI) and evidence of microvascular damage (i.e., no reflow) at 3 ± 2 days from the index event. While baseline regional myocardial perfusion was similar between the two groups, only TTS patients demonstrated rapid improvement in WMSI, wall motion defect extent, and LV ejection fraction within 90 seconds of adenosine infusion, with prompt return to baseline after cessation of the infusion. These findings strongly support acute coronary microvascular constriction as a key pathogenetic mechanism in TTS.

Whether microvascular dysfunction may represent the cause or the consequence of TTS has been largely debated. Patel *et al*. [55] studied microvascular reactivity to ACh in 10 patients with a prior history of TTS. Most patients had microvascular dysfunction (frequently severe) with greater vasomotor dysfunction in the microcirculation as compared to the large epicardial arteries, hence suggesting a potential primary role of CMD in the pathophysiology of TTS. Additional support for a causal role of microvascular dysfunction derives from experimental data by Dong and colleagues, who managed to induce TTS by giving physical stress to a murine model genetically knocked out for the Kv1.5 channel, a condition that mimics the CMD phenotype [56]. Of note, TTS was found to be associated with abnormalities in myocardial perfusion that normalized in parallel with the complete recovery of LV function.

These features may help explain why TTS is more prevalent in women, particularly in the post-menopausal period, since estrogen depletion is a predisposing risk factor to catecholamine sensitivity and microvascular reactivity [57]. Indeed, estrogens usually exert cardioprotective effects by attenuating catecholamine toxicity and preserving endothelial function; their reduction may therefore enhance β-adrenergic receptor sensitivity, lower protection to oxidative stress and impair microvascular vasodilatory capacity.

Several small studies proved the feasibility and safety of invasive assessment of absolute coronary blood flow and microvascular resistance using the saline bolus thermodilution method or continue saline infusion thermodilution method. Belmonte *et al*. [58] studied 6 patients affected by TTS with bolus and continuous thermodilution, which showed concordant findings consistent with CMD: additionally, three patients underwent physiological assessment at follow up and two of them showed resolution of microvascular dysfunction.

Whether invasive physiological assessment could identify subgroups of patients at higher risk or guide targeted therapeutic strategies is yet to be determined and warrants further investigation. A large observational clinical trial (NCT06669962) is currently recruiting patients with TTS at presentation to systematically assess the prevalence of CMD and its correlation with clinical phenotype and prognosis.

## 5. Arrhythmogenic Right Ventricular Cardiomyopathy

Arrhythmogenic right ventricular cardiomyopathy (ARVC) is a non-ischemic cardiomyopathy characterized by histological fibro-fatty myocardial replacement and is frequently associated with life-threatening arrhythmias and SCD in young individuals [59].

Over the years, growing evidence has suggested a potential pathogenetic role for microvascular dysfunction in ARVC, although this aspect is to date less studied than in other cardiomyopathies such as HCM or DCM.

Limited evidence dates back to 2011 from a German group that prospectively compared the microvascular dysfunction of patients with non-failing ARVC with healthy controls through PET imaging [60]: while resting MBF was not significantly different between the two groups, ARVC patients exhibited a markedly blunted hyperaemic MBF response, resulting in up to a 50% reduction in CFR and a concomitant increase in coronary vascular resistance.

The pathophysiological rationale was mainly attributed to the impairment of the vasodilatory responsiveness of the arteries primarily driven by abnormal sympathetic myocardial innervation [61]. Notably, ARVC myocardium exhibits an impaired function and regional reduction of the presynaptic norepinephrine transporter (uptake-1), leading to elevated concentrations of synaptic norepinephrine and subsequent downregulation of postsynaptic β-adrenergic receptor density [62].

These autonomic alterations would also lead to an impairment of microcirculation function: attenuation of β-adrenergic-mediated vasodilation during sympathetic activation, combined with preserved α-adrenergic–mediated vasoconstriction, results in reduced maximal MBF during pharmacological stress in ARVC patients [63].

Microvascular dysfunction and sympathetic denervation seem to occur early in the course of the disease process, preceding the development of overt functional abnormalities or fibrosis detectable by CMR or echocardiography, and may contribute to the arrhythmogenesis in ARVC. Nonetheless, data on the possible prognostic implications of CMD in ARVC remain lacking so far.

## 6. Infiltrative and Storage Disorders 

Infiltrative cardiomyopathies may occur not only with myocardial tissue alterations, which lead to mechanical dysfunction and progression to biventricular HF, but also with significant coronary microvascular remodelling and dysfunction. In this context, CMD can contribute to symptoms like chest pain and shortness of breath, potentially worsening overall prognosis of these cardiomyopathies.

Among infiltrative disorders, amyloidosis and Anderson–Fabry disease are most consistently associated with CMD, whereas evidence for microvascular involvement in inflammatory cardiomyopathies—particularly cardiac sarcoidosis—remains limited [64].

### 6.1 Cardiac Amyloidosis 

Cardiac amyloidosis (CA) is characterized by the extracellular deposition of insoluble fibrils composed of misfolded proteins. More than 40 recognized human proteins are known to form amyloid deposits, and amyloidosis is classified according to the protein precursor: free light chain (AL) and misfolded transthyretin types are the most common forms of cardiac amyloidosis, with vascular involvement being more pronounced in AL amyloidosis. In this subtype, extensive interstitial infiltration by circulating free light chains leads to amyloid fibril accumulation, either diffusely surrounding myocytes or forming nodular aggregates. These deposits are preferentially located in the subendocardial and mid-wall myocardial regions. Amyloid disrupts extracellular matrix homeostasis by impairing matrix metalloproteinase regulation, promoting tissue remodelling, myocyte atrophy, and ultimately replacement fibrosis.

While epicardial coronary arteries may show amyloid infiltration - most commonly in the adventitial layer - clinically significant luminal obstruction is rare. Conversely, intramural coronary microvasculature is frequently detected in AL amyloidosis, with up to 90% of patients demonstrating amyloid deposits, typically in the tunica media and in the intima with disease progression, occasionally resulting in complete luminal occlusion. Such microvascular compromise contributes to focal ischemia, microinfarction, and progressive myocardial fibrosis, further exacerbating LV dysfunction. At the cellular level, light chains induce oxidative stress in both cardiomyocytes and endothelial cells, impairing endothelial-dependent vasodilation through increased generation of reactive oxygen species [65].

Overall, CMD in CA arises from a multifactorial interplay of structural microvascular infiltration, endothelial and autonomic dysfunction, and extravascular compression due to interstitial amyloid deposition, collectively impairing myocardial perfusion and ventricular mechanics.

Historically, CMD in AL amyloidosis has been inferred from functional imaging studies showing stress-induced wall motion abnormalities in the absence of epicardial CAD. Reduced CFR has been documented using intracoronary Doppler techniques [66].

More recently, PET imaging with ^13^NH_3_ has enabled quantitative assessment of MBF: in symptomatic patients without epicardial CAD, MBF and CFR were significantly reduced, independently of LV mass or amyloid subtype, likely reflecting regional heterogeneity in tissue composition [67].

CMR further supports these findings: T1 mapping and LGE detect interstitial expansion, while first-pass perfusion imaging reveals regional hypoperfusion correlating with LV systolic dysfunction [68, 69].

### 6.2 Anderson-Fabry Disease

Anderson-Fabry disease (AFD) is an X-linked lysosomal storage disorder caused by α-galactosidase A deficiency, leading to glycosphingolipid accumulation and multiorgan involvement. The classical form typically presents in childhood or adolescence, predominantly in males, with near-complete enzyme deficiency and early systemic manifestations including neuropathic pain, angiokeratomas, hypohidrosis, renal dysfunction, and early cardiac involvement. In contrast, the non-classical (late-onset) form is characterized by residual enzyme activity and a later presentation, often with predominant single-organ involvement, most commonly cardiac, renal, or cerebrovascular. Cardiac manifestations occur in 40–60% of patients and include arrhythmias, angina and dyspnoea. Glycolipid deposition in myocardial tissue, conduction systems, endothelium and valves results in progressive myocyte hypertrophy and fibrosis, producing a hypertrophic phenotype. In this context CMD is driven by endothelial dysfunction, NO dysregulation and microvascular remodelling, and is increasingly recognized [70].

PET imaging studies have shown reduced hyperaemic MBF, decreased CFR and elevated coronary resistance in AFD patients, even in the absence of left ventricular hypertrophy (LVH). CMD has been documented irrespective of gender and LV hypertrophy status, affecting both males and heterozygous females, the latter often manifesting cardiac symptoms later in life [71].

Multiparametric CMR imaging corroborates these findings, demonstrating reduced MBF in the early phases of the disease, prior to structural myocardial changes such as LVH or fibrosis [72, 73]. Notably, CMD appears to precede even the detectable storage phase marked by low native T1 values [74].

Indeed, available evidence suggests that CMD may be the earliest detectable sign of cardiac involvement in AFD. Thus, assessing microvascular function using advanced imaging modalities may offer critical insights for early diagnosis and therapeutic intervention. Early identification of CMD could support prompt initiation of disease-specific therapies, such as enzyme replacement therapy or pharmacological chaperones (migalastat), potentially altering disease progression and improving clinical outcomes [70].

### 6.3 Sarcoidosis

Sarcoidosis is a systemic inflammatory disorder marked by the formation of non-caseating granulomas in genetically predisposed individuals. Although primarily affecting pulmonary structures, cardiac involvement occurs in 5–10% of patients and can lead to severe complications [75]. Granulomatous infiltration typically affects the LV free wall, followed by the septum, right ventricle, and atria, leading to myocyte injury and replacement fibrosis, while the epicardial coronary arteries are usually spared.

Evidence from PET imaging studies [76] demonstrates that concurrent myocardial perfusion and metabolic abnormalities significantly increase the risk of cardiac death and ventricular arrhythmias. CMD has emerged as a pivotal mechanism in this context, mainly driven by systemic inflammation. Kruse *et al*. [77] demonstrated that regions with abnormal 18-fluoro fluorodeoxyglucose (^18^F-FDG) uptake exhibit impaired hyperaemic MBF and CFR, accompanied by increased coronary resistance. Notably, perfusion deficits can extend to FDG-normal regions in advanced disease, suggesting a diffuse, functional microvascular impairment preceding structural myocardial alterations.

Importantly, immunosuppressive therapy appears to preserve microvascular function in responders, whereas non-responders exhibit further CFR deterioration [77]. Hybrid imaging techniques combining CMR and ^18^F-FDG PET allow for stage-specific characterization (inflammation, necrosis, fibrosis) [78]. Furthermore, CMD in cardiac sarcoidosis is attributed to inflammatory cytokine-mediated endothelial dysfunction, particularly involving TNF-α and oxidative stress pathways, resulting in reduced NO bioavailability and impaired vasodilation.

Emerging evidence also supports the role of second-line invasive physiological tests, such as the assessment of IMR in case of strong suspicion of CMD with negative CMR results [79].

Patients with sarcoidosis, even in the absence of known cardiac involvement or traditional cardiovascular risk factors, show reduced myocardial flow reserve compared to healthy controls. Overall, CMD in this case must be considered as multifactorial, with early microvascular inflammation preceding overt myocardial damage, underlining the importance of early detection and therapeutic modulation of inflammatory activity.

### 6.4 Coronary Microvascular Dysfunction and Energetic Failure: The “Engine Out of Fuel” Paradigm

Across all these cardiomyopathic conditions, a unifying mechanism emerges: the failing heart can be conceived as an “engine out of fuel”, where coronary microvascular dysfunction (CMD) accelerates a profound bioenergetic crisis.

In the early stages, the metabolic shift away from fatty acid oxidation towards alternative substrates is intended as an adaptive strategy to preserve adenosine triphosphate (ATP) production [80]. Yet, this shift comes at the cost of reduced availability of metabolic by-products that normally act as vasodilatory stimuli, such as adenosine or nitric oxide signalling. As these signals decline, the coronary microcirculation progressively loses its ability to adjust flow to demand: coronary flow reserve falls, while the index of microvascular resistance rises, reflecting a stiff and unresponsive microvascular bed [81].

At the same time, mitochondrial dysfunction undermines the efficiency of oxidative phosphorylation, so that the myocardium produces less ATP precisely when oxygen delivery becomes more limited. The consequence is a self-perpetuating cycle: reduced ATP generation blunts vasodilatory signalling, which increases IMR and decreases CFR, further limiting perfusion, worsening energy deficit, and amplifying contractile dysfunction [82].

From this perspective, CMD is not a mere epiphenomenon of cardiomyopathies, but rather the pathophysiological bridge between metabolic remodelling and the progression of HF. The heart becomes an engine running increasingly on empty, less efficient, poorly perfused, and trapped in an energetic downward spiral.

## 7. Treatment and Ongoing Studies 

### 7.1 CMD Treatment

Given the emerging crucial role of microvascular circulation in the most common non-ischemic cardiomyopathies, recent studies have tried to find therapeutic strategies targeting CMD. Unfortunately, to date scarce evidence is available on specific medications focused on the pathophysiology of microvascular dysfunction. However, the first step is always the optimization of cardiovascular risk factors control, since physical training, weight loss and smoking cessation have proven beneficial in improving CFR and endothelial-dependent vasodilatation [83, 84, 85]. Regarding useful medications, statin therapy in dyslipidemic patients has been shown to increase CFR due to its pleiotropic anti-oxidant and anti-inflammatory effect [86]. Likewise, both β-blockers, notably the third-generation class with vasodilating properties (i.e., nebivolol or carvedilol), and dihydropyridine calcium-channel blockers may modify endothelial function, reduce myocardial oxygen demand and increase diastolic perfusion time [87]. Angiotensin-converting enzyme inhibitors were proven beneficial in reversing endothelial dysfunction in the TREND trial [88], whereas the ongoing PRISTINE trial (NCT04128891) will shed further light on the potential benefits of sacubitril/valsartan on CMD.

Other antianginal drugs, such as ivabradine, ranolazine, nicorandil and trimetazidine have been previously studied with controversial results: if nitrates and ivabradine were able to improve angina without significant impact on microvascular function, ranolazine showed a weak non-significant positive effect on CFR, albeit in limited number cohorts [87, 89].

Finally, also sodium glucose cotransporter 2 inhibitors (SGLT2i) have shown beneficial effects on CMD, given their effect on mediators of microvascular pathophysiology, such as cytokines, inflammatory mediators, vascular smooth cell proliferation and endothelial dysfunction [90]. Indeed, in preclinical mice models, SGLT2i improved CFR and fractional area change [91], while clinical data are controversial with only limited benefit in this sense [92, 93]. Other agents with theoretical benefit but insufficient evidence to support efficacy include L-arginine, phosphodiesterase-5 inhibitors and adenosine-receptor antagonists (i.e., aminophylline or caffeine).

Further nonpharmacological treatments are currently being studied: CD34+ cell therapy, transcutaneous electrical nerve stimulation, spinal cord stimulation and external counter pulsation have shown promising results despite the limited available evidence [94]. In this context, coronary sinus reducer deserves a separate discussion, as much interest has raised to identify a potential benefit of this device to improve CFR. Although the recent ORBITA-COSMIC trial failed to demonstrate an improvement of myocardial perfusion after reducer implantation [95], further evidence is coming from the ongoing REMEDY-PILOT (Reducing Microvascular Dysfunction in Patients With Angina, Ischaemia and Unobstructed Coronary Arteries, NCT05492110) and COSIMA (Coronary Sinus Reducer for the Treatment of Refractory Microvascular Angina, NCT04606459) trials [96].

### 7.2 Ongoing Studies on Invasive CMD Assessment in Cardiomyopathies

The emerging role of invasive assessment of CMD has led to an increasing number of studies on microvascular disease in several contexts, performed by interventional cardiologists.

The international MICROREV-DCM project (Microvascular Dysfunction Assessment to Predict Left Ventricular Reverse Remodeling, NCT06356727) aims to use invasive IMR and CFR in patients with newly diagnosed idiopathic DCM to predict which patients will experience improvement in ventricular remodelling with therapy during follow-up. The results of this study may clarify the clinical utility of early invasive microcirculation assessment in the management of non-ischemic heart failure.

The Korean group of Joo Myung Lee and colleagues has started a trial (Physiologic Assessment of Microvascular Function in Patients with Cardiac Amyloidosis, NCT02798705) aiming to evaluate CFR and IMR in CA to assess the role of microvascular disease in this disease. Additionally, this study will evaluate the association between physiologic indices and pathologically measured percent area involvement of interstitium as well as the correlation between the invasive and non-invasive measurements in this context.

Finally, another prospective multicentre registry is the REDUCE-CMD (micRovascular and EpicarDial invasive evalUation in patients with reduCed ejEction fraction CardioMyopathy) which aims to evaluate microvascular and epicardial physiology in patients with reduced left ventricular ejection fraction (<50% at echocardiogram or CMR) and intermediate coronary stenosis. This study is not focused on a specific type of cardiomyopathy but focuses on the role of microvascular circulation and physiology assessment in the setting of reduced left ventricular ejection fraction with concomitant subcritical CAD.

## 8. Conclusions

**Fig. 2.**
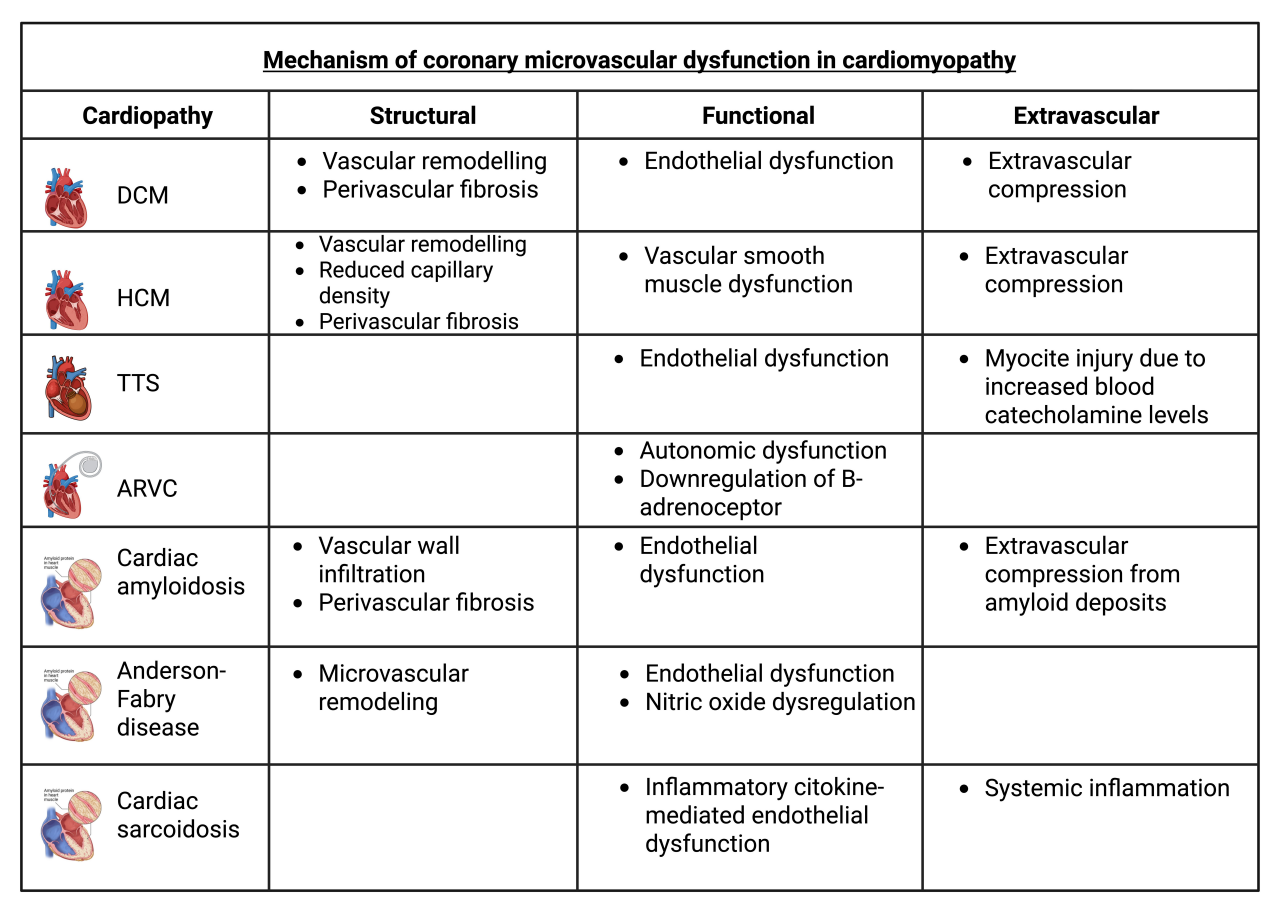
**Coronary Microvascular Dysfunction mechanisms across the different cardiomyopathies**. Created with BioRender.

Coronary microvascular dysfunction represents a central and previously underappreciated component across a broad spectrum of non-ischemic cardiomyopathies. Although the underlying mechanisms of such disease vary depending on the specific phenotype (Fig. 2), a common pathophysiological theme emerges: impairment of the coronary microcirculation acts as a key mediator linking myocardial injury, adverse remodelling, and clinical progression. In HCM, microvascular dysfunction is common and acts as a mediator of ischaemia, fibrosis, disease progression and represents an independent risk factor for SCD and left ventricular function impairment. In DCM, CMD reflects widespread structural impairment of the myocardium and is associated with worse prognosis and a reduced likelihood of functional recovery. In arrhythmogenic cardiomyopathy (ACM)/arrhythmogenic right ventricular cardiomyopathy (ARVC), emerging evidence suggests stress-induced hypoperfusion and early microvascular involvement, although the prognostic implications of CMD in this setting remain to be fully elucidated. In infiltrative cardiomyopathies, particularly amyloidosis, microvascular impairment is ubiquitous and contributes substantially to anginal symptoms and contractile dysfunction; quantitative perfusion parameters (PET or CMR) have demonstrated strong additional prognostic value. From a practical standpoint, the focus on microcirculation in cardiomyopathies is leading to new integrated diagnostic approaches.

**Fig. 3.**
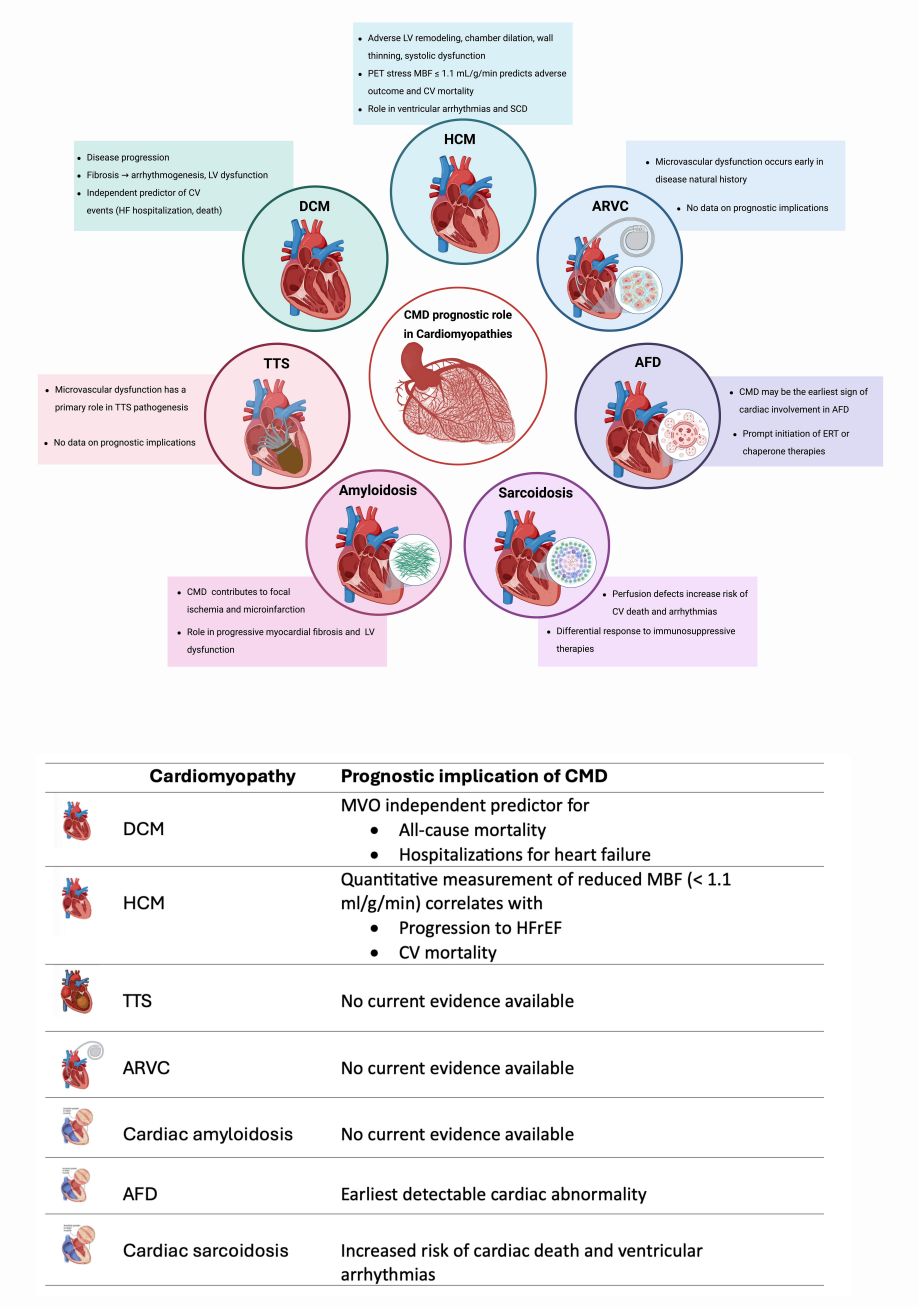
**Prognostic role and implication of coronary microvascular dysfunction in cardiomyopathies**. Created with BioRender.

Beyond pathophysiological insights, increasing recognition of CMD is reshaping the diagnostic and prognostic approach to cardiomyopathies. Contemporary guidelines, including the 2023 ESC Guidelines on cardiomyopathies, emphasize the importance of a multimodal evaluation integrating advanced cardiac imaging—such as CMR with tissue characterization and quantitative perfusion techniques—with functional assessment of myocardial blood flow. In this context, the identification of CMD provides clinically meaningful information for risk stratification, follow-up intensity, and patient phenotyping (Fig. 3).

Looking forward, CMD should no longer be regarded as a secondary epiphenomenon but rather as a dynamic and potentially modifiable disease component. Future research should aim to standardize diagnostic criteria, define CMD-related risk thresholds, and explore targeted therapeutic strategies aimed at improving microvascular function. Ultimately, incorporating systematic assessment of the coronary microcirculation into the routine evaluation of cardiomyopathy patients may represent a critical step toward more personalized risk stratification and disease-modifying interventions.
